# Increased IR-A/IR-B ratio in non-small cell lung cancers associates with lower epithelial-mesenchymal transition signature and longer survival in squamous cell lung carcinoma

**DOI:** 10.1186/1471-2407-14-131

**Published:** 2014-02-26

**Authors:** Liyan Jiang, Wei Zhu, Katie Streicher, Chris Morehouse, Philip Brohawn, Xiaoxiao Ge, Zhengwei Dong, Xiaolu Yin, Guanshan Zhu, Yi Gu, Koustubh Ranade, Brandon W Higgs, Yihong Yao, Jiaqi Huang

**Affiliations:** 1 Department of Pulmonary, Shanghai Chest Hospital, Shanghai Jiao Tong University, Shanghai, China; 2 MedImmune Inc., LLC, One MedImmune Way, 20878 Gaithersburg, MD, USA; 3 Innovation Center China, AstraZeneca Global R&D, Shanghai, China

## Abstract

**Background:**

To evaluate the insulin receptor isoform mRNA expression status in non-small cell lung cancer (NSCLC) patients.

**Methods:**

RNA-seq data from 614 NSCLC [355 adenocarcinomas (LUAD) and 259 squamous cell carcinomas (LUSC)] and 92 normal lung specimens were obtained from The Cancer Genome Atlas (TCGA) to evaluate the mRNA expression of insulin receptor isoform A (IR-A) and insulin receptor isoform B (IR-B). The differential expression status of the insulin receptor isoforms in NSCLC patients was confirmed using qRT-PCR assays with lung cancer cDNA arrays and primary tumor samples.

**Results:**

The mRNA expression levels of IR-B were significantly lower in some NSCLC samples compared to normal lung specimens, including both LUAD and LUSC. Notably, no IR-B transcripts were detected - only the IR-A isoform was expressed in 11% of NSCLC patients. This decrease in IR-B expression contributed to an elevated IR-A/IR-B ratio, which was also associated with lower epithelial-mesenchymal transition gene signatures in NSCLC and longer patient survival under standard of care in LUSC. In addition to NSCLC, RNA-seq data from TCGA revealed a similar increase in IR-A/IR-B ratio in many other cancer types, with high prevalence in acute myeloid leukemia, glioblastoma multiforme, and brain lower grade glioma.

**Conclusions:**

Our results indicate a common reduction of the mRNA expression level of IR-B and an increased IR-A/IR-B mRNA ratio in NSCLC and other tumor types. The relationship of altered IR-A/IR-B ratios with cancer progression and patient survival should be prospectively explored in future studies.

## Background

Lung cancer is the leading cause of cancer death and the second most diagnosed cancer in both men and women in the U.S. In 2008, 14% of all cancer diagnoses and 28% of all cancer deaths were due to lung cancer [1]. Non small-cell lung cancer (NSCLC) is the most common type of lung carcinoma and accounts for at least 85% of all lung cancer cases in the US [2]. Adenocarcinomas (LUAD) and squamous cell carcinomas (LUSC) are the most common subtypes of NSCLC.

Insulin is a crucial growth factor that binds specifically to the insulin receptor (INSR) and subsequently activates the PI3K-AKT pathway. This pathway is mainly responsible for mediating the metabolic effects of insulin and regulating the MAP kinase pathway that influences important biological processes, such as cell growth and differentiation [3]. The mature human INSR has two isoforms: Insulin receptor isoform A (IR-A) and Insulin receptor isoform B (IR-B), which form from alternative splicing of the same primary transcript [4]. The biological roles of IR-A and IR-B are different. IR-B is a classical insulin receptor which only binds to insulin, while IR-A has high affinities to both insulin and IGF2. IR-B is responsible for the major metabolic effects of insulin in muscle, liver, and adipose tissues. IR-A promotes growth and anti-apoptotic effects under physiological conditions like embryonic development [4,5]. The relative level of mRNA encoding IR-A and IR-B is regulated not only in a tissue-specific manner [4,5], but also depends on the stage of cell development and differentiation. For example, in fetal tissues and cancerous cells, IR-A is the predominant isoform [5].

Dysregulation of the INSR has been reported in multiple cancers [6-8]. INSR over-expression has also been associated with lung tumor progression [4,9]. Since distinct biological roles of IR-A and IR-B exist, it is important to evaluate the relative abundance of IR-A and IR-B expression in NSCLC and evaluate their associated prognostic values.

In this study, we analyzed RNA-seq data from 614 NSCLC (355 LUAD and 259 LUSC) and 92 normal lung tissues from The Cancer Genome Atlas (TCGA). We observed that IR-B mRNA expression was significantly lower in some NSCLC specimens (both LUAD and LUSC) compared to adjacent normal lung tissues, thereby contributing to altered IR-A/IR-B mRNA ratio in this disease. Intriguingly, we observed that patients with higher IR-A/IR-B mRNA ratio generally showed upregulated oxidative phosphorylation pathway, lower epithelial-mesenchymal transition (EMT) gene expression signatures in NSCLC and exhibited longer survival under standard of care in LUSC. Additionally, the down regulation of IR-B and higher IR-A/IR-B mRNA ratio was also displayed in other 18 tumor types. Overall, our results suggest that the IR-A/IR-B mRNA ratio may serve as a prognostic maker to guide clinical treatment decisions of LUSC; and characterizing the specific relationship of this biomarker with prognosis and treatment response might also be valuable for other cancer indications.

## Methods

### Molecular profiling and data processing

Normalized expression data of the genes and transcript isoforms for NSCLC were downloaded from Level 3 RNA-seq data of TCGA (https://tcga-data.nci.nih.gov/tcga/dataAccessMatrix.htm) dated March, 2013. The data include both LUAD and LUSC. TCGA collection includes 614 treatment naive NSCLC tumor samples (355 LUAD and 259 LUSC) and 92 adjacent normal lung tissues. All cancer specimens are comprised of at least 75% tumor tissue.

### Bioinformatics analysis of insulin receptor isoforms using RNA-seq data

The normalized isoform expression files and exon expression files generated by TGCA provide expression information of INSR isoforms. IR-A and IR-B mRNA expression levels were retrieved from normalized isoform expression files. The expression levels of IR-A and IR-B mRNA were compared between normal lung tissues and tissues from LUSC and LUAD. The mRNA expression ratio of IR-A and IR-B was calculated using RNA-Seq by Expectation-Maximization (RSEM) normalized read counts (https://wiki.nci.nih.gov/display/TCGA/RNASeq); for 0 read counts and the others as well, evaluation of the distribution justified a pseudo count of 0.1. The IR-A/IR-B ratios between LUSC, LUAD and adjacent normal tissue were compared using Wilcoxon-Mann–Whitney two group (two-tailed) test. Since IR-A differs from IR-B by the exclusion of exon 11, we also calculated IR-A/IR-B ratios using the exon 10, 11, and 12 normalized expression files from TCGA to quantify the expression levels of IR-A and IR-B for the quality assurance.

### Experimental confirmation of INSR isoform expression status in NSCLC

Experimental confirmation of INSR isoform expression status was performed in two independent panels of NSCLC specimens. Panel 1 consists of five lung cancer cDNA arrays (HLRT101, HLRT102, HLRT103, HLRT104, and HLRT105) purchased from OriGene Technologies (Rockville, MD). The arrays contained cDNAs from 50 normal lung tissue (38 unique donors), 84 adenocarcinoma and 60 squamous-cell carcinoma samples. The tumor stage ranged from Stage IA to IV. The tumor samples were comprised of 35-95% tumor. Panel 2 included primary, fresh frozen, treatment naïve NSCLC tumors from 24 patients and adjacent normal tissues from 12 of the 24 patients collected from the Shanghai Chest Hospital. Each tumor sample was comprised of greater than 70% tumor. All patients provided written informed consent before study-related procedures were performed.

The primers and probes of TaqMan gene expression assays for IR-A and IR-B and methods were described in detail in Huang et al. [10]. The reference genes ACTB (Hs99999903_m1), GAPDH (Hs99999905_m1) and GUSB (AssyID: Hs99999908_m1) were purchased from Life Technologies.

cDNA samples in panel 1 were preamplified using TaqMan Pre-Amp Master Mix (Life Technologies, CA), according to the manufacturer’s instructions. Reactions contained 5 μL of cDNA, 10 μL of Pre-Amp Master Mix, and 5 μL of 0.2× gene expression assay mix (comprised of all primer/probes to be assayed) at a final reaction volume of 20 μL. Reactions were cycled with the recommended 14-cycle program and then diluted 1:5 with TE buffer. Preamplified cDNA was used immediately or stored at −20°C until processed. For PCR, the BioMark RT-PCR System (Fluidigm, CA) was utilized as previously described [10].

The number of replicates and the composition of the samples varied depending on the particular experiment but were never less than triplicate. Average Cycle Threshold (Ct) values were used to determine sensitivity and specificity of the designed probes. Ct values were extracted from each assay with the SDS v2.0 software tool (Applied Biosystems, CA). The average Ct values of all available reference gene assays within a sample were utilized for calculation of ΔCt.

MRNA for panel 2 samples was isolated using Ambion Recover All Total Nucleic Acid Isolation kit (Life Technologies). RNA quality was assessed on an Agilent 2100 Bioanalyzer using the RNA 6000 Nano LabChip® (Agilent technologies, CA). RNA purity and concentration were determined spectrophotometrically(260/280 > 1.9). 2ug RNA were reverse transcribed to cDNA following the manufacturer’s protocol. The same volume of cDNA for each sample was evaluated by qPCR.

All samples were normalized to the average expression levels of the 3 housekeeping genes: ACTB, GAPDH and UBC. The relative expression level was represented by -ΔCt, where -ΔCt = − [(Ct of a gene of interest) - (Average Ct of 3 housekeeping genes)].

The relative difference in expression level between tumor and normal lung samples was represented by -ΔCt, and the IR-A/IR-B expression ratio was presented as the ΔCt differential (IR-A ΔCt – IR-B ΔCt). To determine over-expression of genes in lung cancer relative to normal lung, we calculated fold changes values using the formula 2 ^-ΔΔCt^, where ΔΔCt for a gene of interest is defined as: (ΔCt lung cancer sample - mean ΔCt of all normal samples). A cutoff of 2-fold was used to determine over-expression.

### Molecular characterization of NSCLC tumors with higher IR-A/IR-B ratio

Based on the distribution of IR-A/IR-B ratio, the tumor samples were divided into two groups: high IR-A/IR-B ratio (HIR) and low IR-A/IR-B ratio group (LIR). The genes with significant differential expression (fold change > 2, Benjamini-Hochberg adjusted p-value < 0.05) were identified between the two groups, in order to identify gene expression signatures associated with a high IR-A/IR-B ratio.

### Clinical characterization of squamous cell carcinoma (LUSC) patients with higher IR-A/IR-B ratio

Clinical information for LUSC samples were downloaded from TCGA (Level 2 Biolab data). The clinical features of patients with HIR were compared to patients with LIR. The survival analyses were conducted using R (http://www.r-project.org). The median overall survival (OS) was determined using the Kaplan-Meier method from the R package *survival*. The Cox regression model was applied to assess IR-A/IR-B ratio on the prognostic value of OS, which was adjusted by patient covariates including gender, smoking history, age at initial pathologic diagnosis, tumor stage, and treatment with chemotherapy. P values < 0.05 were considered statistically significant.

## Results

### Higher IR-A/IR-B mRNA ratio is observed in NSCLC patients using a large patient population from TCGA

**Figure 1:**
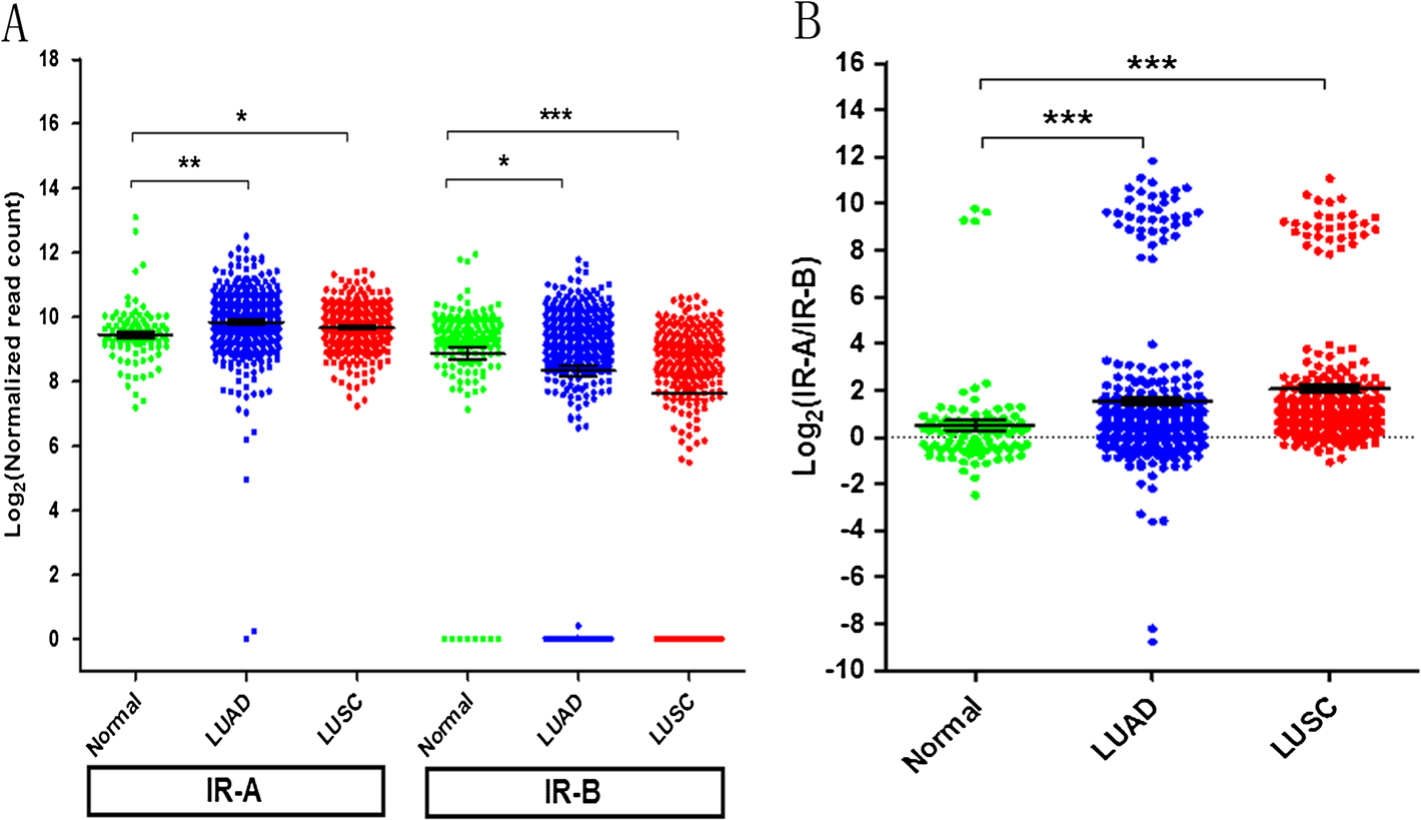
**Transcript abundance of insulin receptor isoforms in LUAD and LUSC profiled by TCGA RNA-seq data. A)** Log_2_-transformed and normalized read count of IR-A and IR-B receptor isoforms in LUAD (n = 355), LUSC (n = 259) and adjacent normal tissue (n = 92) and **B)** the corresponding distribution of log_2_-transformed IR-A/IR-B mRNA ratio. Asterisks indicate statistically significant differences (* = P < 0.05; ** = P < 0.01; *** = P < 0.001).

To evaluate the expression of IR-A and IR-B mRNAs in NSCLC, we utilized the large RNA-seq database from TCGA. As shown in Figure 1A, the median normalized expression of IR-B is statistically significantly lower in LUSC (P < 0.0001) but not in LUAD (P = 0.064) as compared to normal lung. Notably, a small fraction of NSCLC samples (11%) do not express IR-B. The median normalized mRNA expression of IR-A is also significantly higher in LUAD (P = 0.001) and LUSC (P = 0.043) compared to normal lung tissues.

The mRNA ratio for IR-A and IR-B was additionally assessed for both LUAD and LUSC, and results are shown in Figure 1B. The IR-A/IR-B mRNA ratio is statistically significantly higher in LUAD (P < 0.001) and LUSC (P < 0.001) compared to normal lung tissues. IR-B mRNA expression was drastically down-regulated in a fraction of NSCLC tumor samples, as shown by the bimodal distribution of the IR-A/IR-B ratio observed in these samples (Figure 1B). To confirm the results, we also calculated the IR-A/IR-B mRNA ratio using the normalized exon expression values for exons 10, 11, and 12 of INSR from TCGA and similar results were observed (data not shown).

We also assessed the mRNA expression levels of IGF1R and found that 12 out of 144 (8%) NSCLC samples have >2 fold IGF1R mRNA expression than the normal lung samples in the panel 1. We explored relationships between IGF1R and INSR isoforms and uncovered no clear relationships in our test panels or TGCA data sets.

### TaqMan qRT-PCR confirms the decreased mRNA level of IR-B and increased IR-A/IR-B mRNA ratio in NSCLC

**Figure 2:**
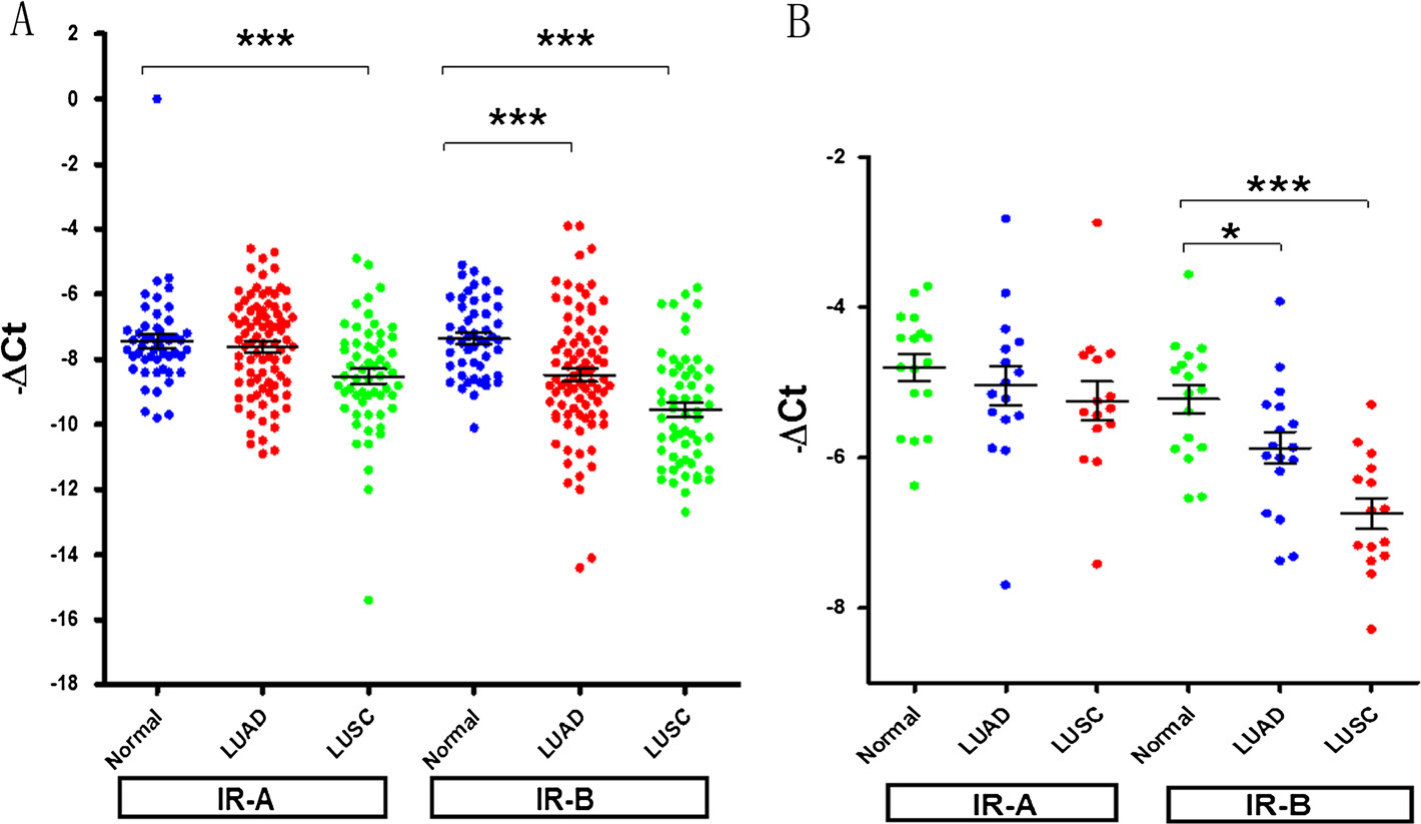
**TaqMan qRT-PCR confirmation of relative mRNA Expression Levels for IR-A and IR-B in NSCLC primary tumors run on cDNA arrays.** TaqMan gene expression assays determined the relative mRNA expression levels of IR-A and IR-B. For each sample, the expression levels of IR-A and IR-B were normalized to the average expression levels of 3 housekeeping genes (ACTB, GAPDH and UBC). The relative expression levels of IR-A and IR-B are represented by -ΔCt, where -ΔCt = − [(Ct of a gene of interest) - (Average Ct of 3 housekeeping genes)]. The error bars represent the mean -ΔCt ± 95% CI within a particular gene target and sample-type combination. **(A)**: data from cDNA array, **(B)** data from primary tumor samples. Asterisks indicate statistically significant differences (* = P < 0.05; ** = P < 0.01; *** = P < 0.001).

The TaqMan qRT-PCR measurements of mRNA expression levels of IR-A in NSCLC using cDNA array (Panel 1; see Methods) and NSCLC primary tissue (Panel 2; see Methods) are shown in Figure 2A and Figure 2B. A two-sample test indicated that the mRNA levels for IR-A were significantly lower in LUSC specimens (P = 0.0007) compared to normal lung specimens from cDNA arrays (Figure 2A), with a similar trend observed in primary tumor specimens (Figure 2B). The mRNA levels for IR-A were not significantly different between normal lung and LUAD specimens (P = 0.050) in either sample set. The mRNA levels for IR-B were significantly lower in LUAD (P < 0.001) and LUSC primary tumor specimens comparing to normal lung tissues (P < 0.001) run on cDNA array (Figure 2). Although there are differences in IR-A mRNA expression in LUAD and LUSC versus normal in TCGA data compared with results from panel 1 and 2, the overall magnitude of changes in IR-A expression are rather modest compared to those in IR-B. Differences in sample size and content of the tumor samples could contribute to the minor variability in IR-A expression observed between these datasets.

**Figure 3:**
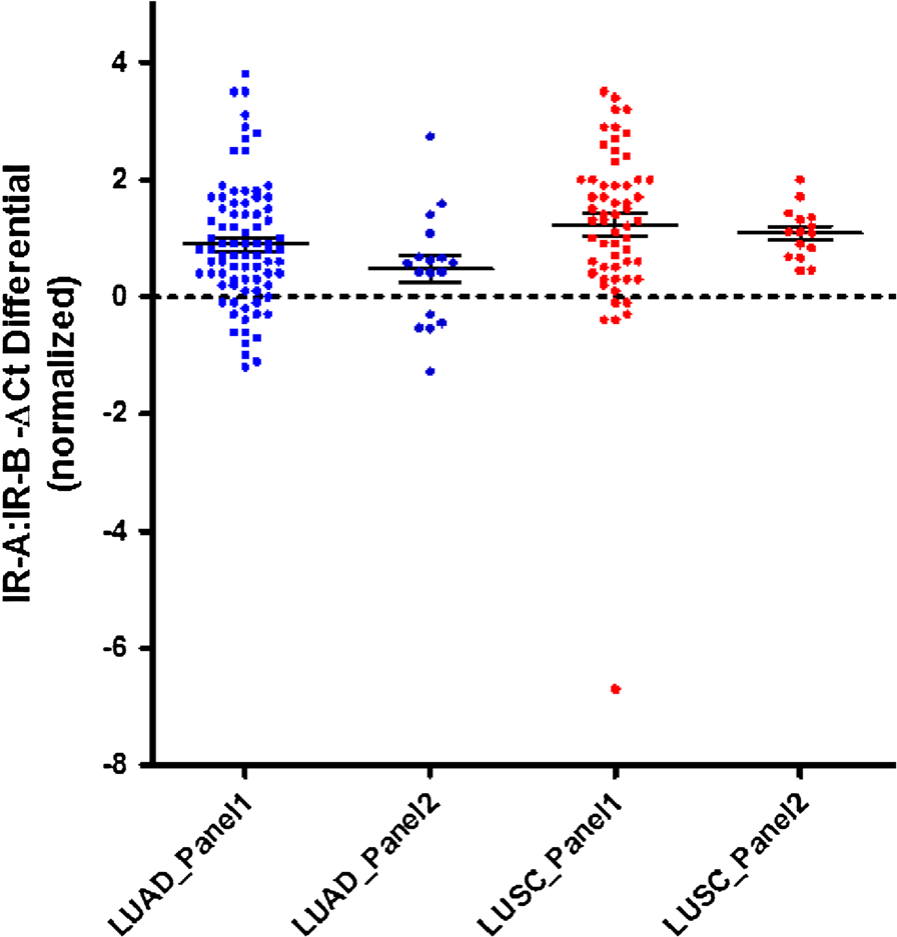
**TaqMan qRT-PCR confirmation of IR-A/IR-B mRNA ratio in NSCLC primary tumors using cDNA array.** The IR-A/IR-B mRNA ratio was assessed by determining ΔCt differentials of IR-A and IR-B in lung cancer and normal lung samples. The ΔCt differential (IR-B ΔCt – IR-A ΔCt) values were calculated for each specimen utilizing the within-specimen reference gene panel (average Ct) and normalized to the average of the normal lung samples. The error bars represent the mean -ΔCt ± 95% CI within a particular gene target and sample-type combination. Dotted line indicates average levels of IR-A/IR-B ratios in normal lung tissues.

IR-A/IR-B mRNA ratios in tumors from panel 1 and panel 2 were normalized to the average IR-A/IR-B mRNA ratio from normal lung specimens and are shown in Figure 3. The mean IR-A/IR-B mRNA ratio was significantly higher in LUAD samples (P < 0.001) and LUSC samples (P < 0.001), compared to normal lung specimens. The increase in IR-A/IR-B mRNA ratio and decreased IR-B mRNA expression observed in primary and cDNA array samples is consistent with what was originally observed using RNA-seq data from TCGA.

### Stratification of patients into IR-A/IR-B mRNA ratio high and low groups and identification of the differentially expressed genes associated with EMT

**Figure 4:**
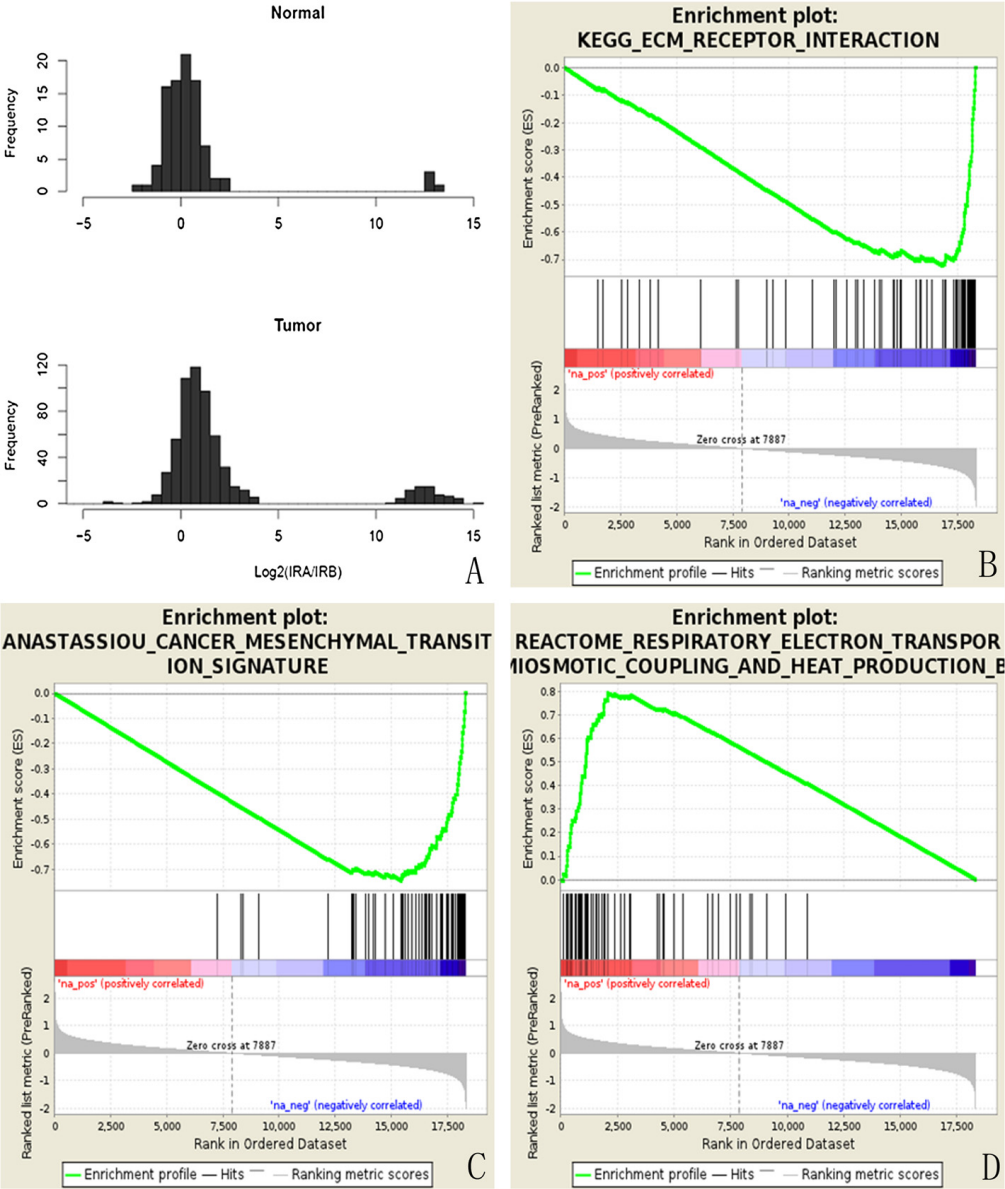
**IR-A/IR-B mRNA ratio in NSCLC and the differentially expressed genes associated with the ratio of IR-A/IR-B mRNA. A.** Distribution of the IR-A/IR-B mRNA ratio among the NSCLC and the adjacent normal lung specimens**.** According to the distribution of IR-A/IR-B mRNA ratio in the adjacent normal lung (the top panel), log_2_(8) (i.e., 3) was selected as the cutoff value to stratify the subjects into two groups: high IR-A/IR-B ratio (HIR) and low IR-A/IR-B ratio (LIR). **B-D**. Gene set enrichment analysis (GSEA) of the differentially expressed genes in NSCLC patients with HIR and LIR. GSEA enrichment plots showing that ECM **(B)** and EMT **(C)** signaling were down-regulated (p < 0.001, respectively) in LUSC samples with HIR. Additionally, the oxidative phosphorylation pathway was highly enriched in the LUSC samples with HIR **(D)**.

To better characterize the molecular landscape of tumors with altered IR-A/IR-B mRNA ratios, we used TCGA data to identify differentially expressed genes in tumors that are associated with high IR-A/IR-B mRNA ratio (HIR). To do this, we evaluated the distribution of the IR-A/IR-B mRNA ratio in NSCLC tumors and normal lung specimens, revealing a bimodal distribution in both sample types (Figure 4A). While most normal samples (88 out of 92) have IR-A/IR-B ratio equal to approximately 1 [log_2_ (mean ± sd) = 0.10 ± 0.84], the distribution in NSCLC patients is shifted, associated with an increased ratio in this cancer type (Figure 4A). Using these distributions, we identified log_2_(8) (i.e., 3, P < 0.001) as the cutoff value, which is at least 3 times the standard deviation from the mean (0.1), to classify the tumor specimens into two groups - high IR-A/IR-B mRNA ratio and low IR-A/IR-B mRNA ratio (LIR). In the subsequent analysis of RNA-seq data, 114 differentially expressed genes shared between LUAD and LUSC were identified (see Methods). Gene set enrichment analysis (GSEA) indicated that the down-regulated genes were significantly enriched with EMT genes (Figure 4C) and genes involved in ECM-receptor interaction (Figure 4B), which have been reported to be significantly increased in invasive NSCLC cancer types (Byers LA, 2013). GSEA also revealed that genes up-regulated in HIR specimens are enriched with genes involved in the mitochondria oxidative phosphorylation pathway (Figure 4D).

At the molecular level, LUSC have been previously divided into four different subtypes (primitive: proliferation; classical: xenobiotic metabolism; secretory: immune response; basal: cell adhesion) which have clinical importance [9]. We utilized this subtype classification generated by Wilkerson et al., to analyze the TCGA data and compare the IR-A/IR-B mRNA ratio among the different subclasses. No significant differences in IR-A/IR-B mRNA ratio were observed (data not shown) among these four subtypes.

### HIR is associated with a better clinical outcome under standard of care

**Figure 5:**
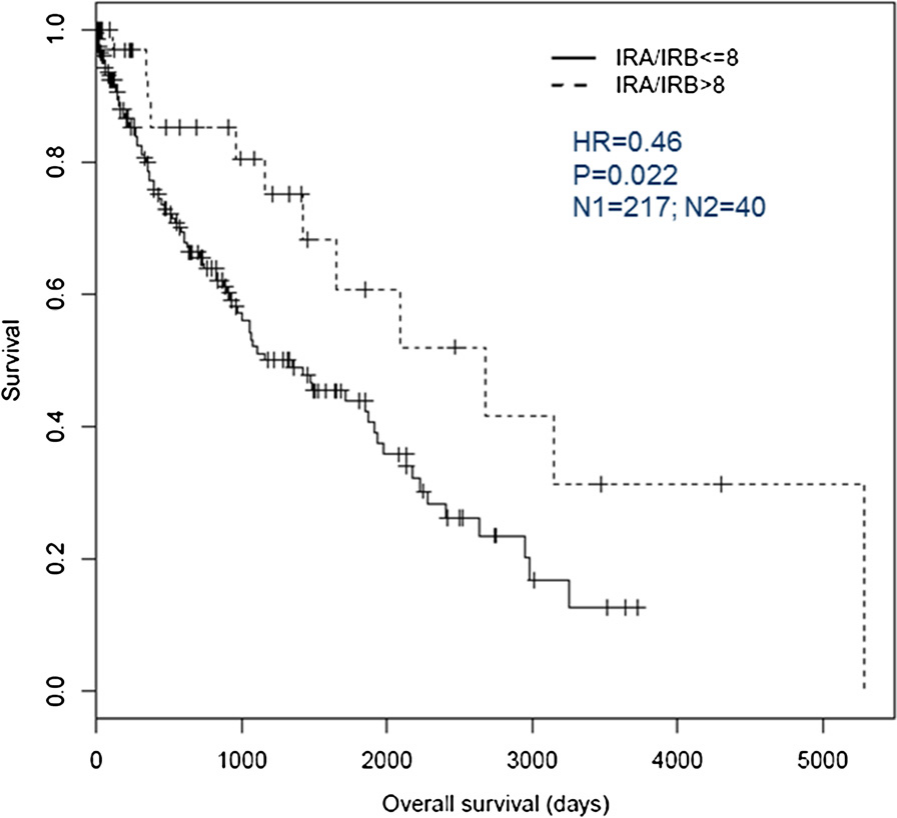
**Kaplan-Meier plot comparing survival in LUSC patients with HIR versus LIR.** Overall survival was adjusted by patient covariates including gender, smoking history, age at initial pathologic diagnosis, tumor stage, and treatment with chemotherapy. A P < 0.05 achieved in the analysis was considered to be statistically significant. The LUSC patients with HIR (dotted line) show a significant difference in survival compared to those with low ratio (dashed line) (hazard ratio, 0.457; 95% CI [0.23, 0.89]; P = 0.022).

The clinical features and survival information of LUSC patients were also utilized from TCGA portal. We evaluated the survival experience associated with IR-A/IR-B mRNA ratio after adjusting for gender, smoking history, age at initial pathologic diagnosis, tumor stage, and chemotherapy treatment. In univariate analysis, no statistically significant association between IR-A/IR-B mRNA ratio and these clinical features was observed. However, the Cox proportional hazards model indicated that patients with HIR have better survival outcome (hazard ratio = 0.46; 95% CI [0.23, 0.89]; P = 0.022; Figure 5), which is also consistent with the association of HIR with a decreased EMT/less invasive phenotype.

### Survey of the IR-A/IR-B mRNA ratio in multiple tumor types

**Figure 6:**
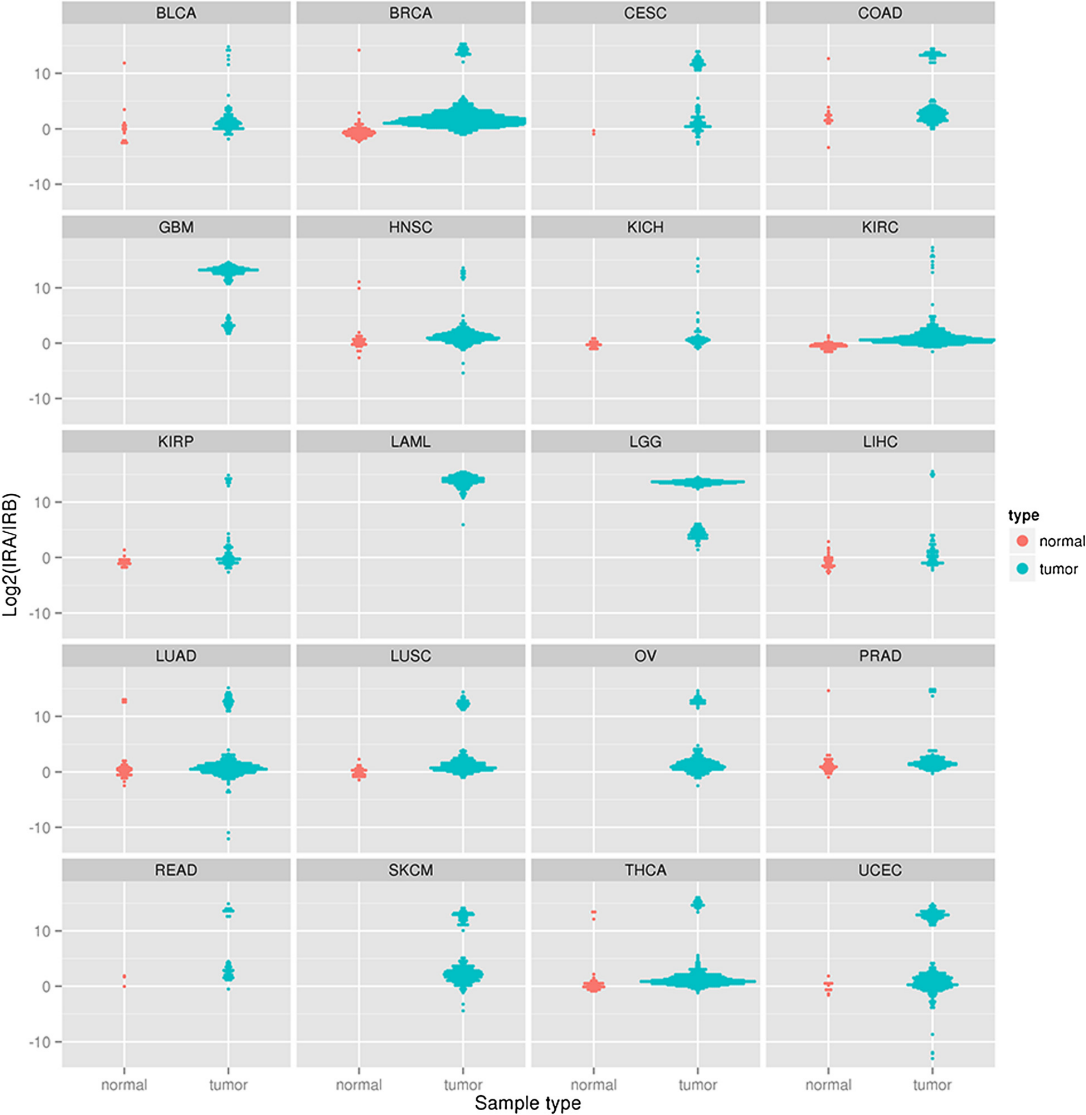
**Distribution of the IR-A/IR-B mRNA ratio in 20 types of cancer evaluated using TCGA RNA-seq database.** According to the distribution of IR-A/IR-B mRNA ratio in the adjacent normal, 8 (=2^3^) is selected as the cutoff value to defined the high IR-A/IR-B ratio group (HIR). Acute Myeloid Leukemia (LAML), Bladder Urothelial Carcinoma (BLCA), Glioblastoma multiforme (GMB), Brain Lower Grade Glioma (LGG), Breast invasive carcinoma (BRCA), Colon adenocarcinoma (COAD), Rectum adenocarcinoma (READ) Head and Neck Squamous Cell Carcinoma (HNSC), Prostate adenocarcinoma (PRAD), Ovarian serous cystadenocarcinoma (OV), Thyroid carcinoma (THCA), Uterine Corpus Endometrioid Carcinoma (UCEC), Kidney Chromophobe (KICH), Kidney renal clear cell carcinoma (KIRC), and Kidney renal papillary cell carcinoma (KIRP) were analyzed for expression levels of IR-A and IR-B and grouped according to HIR or LIR. The numbers of patients in each group are indicated in the table, as well as the fraction of patients with HIR. The P value column indicates a statistically significant difference in the prevalence of HIR in a particular tumor type compared to the associated normal tissue using a binomial proportions test.

To examine whether increased IR-A/IR-B mRNA ratios are a common event occurring in other cancer types, we used TCGA RNA-seq exon and junction level data to assess INSR isoform expression status in tumor and normal adjacent tissues from a panel of cancer types including acute myeloid leukemia (LAML), bladder urothelial carcinoma (BLCA), glioblastoma multiforme (GMB), brain lower grade glioma (LGG), breast invasive carcinoma (BRCA), colon adenocarcinoma (COAD), rectum adenocarcinoma (READ), head and neck squamous cell carcinoma (HNSC), prostate adenocarcinoma (PRAD), ovarian serous cystadenocarcinoma (OV), thyroid carcinoma (THCA), uterine corpus endometrioid carcinoma (UCEC), kidney chromophobe (KICH), kidney renal clear cell carcinoma (KIRC), and kidney renal papillary cell carcinoma (KIRP). The results are shown in Figure 6. A significantly increased IR-A/IR-B mRNA ratio (i.e., >3) was observed in BRCA, COAD, KIRC, KIRP, liver hepatocellular carcinoma (LIHC), and UCEC compared to adjacent normal tissues. It is worth noting that the prevalence of HIR in brain tumors (GMB and LGG) and LAML are greater than 93% (Table 1).

**Table 1:** Statistic summary of the IR-A/IR-B mRNA ratio in 20 types of cancer

**Disease**		**Normal**			**Tumor**		
	**HIR**	**LIR**	**HIR fraction**	**HIR**	**LIR**	**HIR fraction**	P value^*^
Bladder Urothelial Carcinoma (BLCA)	2	14	0.13	16	106	0.13	0.5
Breast invasive carcinoma (BRCA)	1	106	0.01	140	709	0.16	**0**
Cervical squamous cell carcinoma and endocervica adenocarcinoma (CESC)	0	2	0	51	65	0.44	0.3
Colon adenocarcinoma (COAD)	3	15	0.17	90	103	0.47	**0.014**
Glioblastoma multiforme (GBM)	NA	NA	NA	157	12	0.93	NA
Brain Lower Grade Glioma (LGG)	NA	NA	NA	215	5	0.98	NA
Head and Neck squamous cell carcinoma (HNSC)	2	35	0.05	17	286	0.06	0.5
Kidney Chromophobe (KICH)	0	25	0	6	60	0.09	0.139
Kidney renal clear cell carcinoma (KIRC)	0	71	0	27	453	0.06	**0.04**
Kidney renal papillary cell carcinoma (KIRP)	0	25	0	12	64	0.16	**0.039**
Acute Myeloid Leukemia (LAML)	NA	NA	NA	173	0	1	NA
Liver hepatocellular carcinoma (LIHC)	0	36	0	9	60	0.13	**0.029**
Lung adenocarcinoma (LUAD]	4	53	0.07	45	310	0.13	0.158
Lung squamous cell carcinoma (LUSC)	0	35	0	42	218	0.16	**0.01**
Ovarian serous cystadenocarcinoma (OV)	N/A	N/A	N/A	48	218	0.18	NA
Prostate adenocarcinoma (PRAD)	2	42	0.05	12	164	0.07	0.418
Rectum adenocarcinoma (READ)	0	3	0	23	29	0.44	0.182
Skin Cutaneous Melanoma (SKCM)	NA	NA	NA	87	180	0.33	NA
Thyroid carcinoma (THCA)	3	53	0.05	41	373	0.1	0.197
	0	11	0	102	268	0.28	**0.046**

The numbers of samples classified as high IR-A/IR-B mRNA ratio (HIR) and low IR-A/IR-B mRNA ratio (LIR) in each tumor type as well as the statistic significances (P value) of the prevalence of HIR samples in each tumor type compared with that in the corresponded normal tissues are indicated in table.

^*^Bold font indicates statistically significant results i.e., p-value < 0.05.

## Discussion

Preclinical studies suggest that signaling via the INSR isoforms IR-A and IR-B may be of critical importance in NSCLC [11,12]. After the failures of two large randomized phase III anti-IGF1R trials in NSCLC, (Figitumumab [13], hR1507 [13,14]), IR-A signaling has been postulated as one of the major mediators of resistance to anti-IGF1R therapy [11,15]. However, due to technical difficulties, a key measurement to address this issue, the mRNA expression status of insulin receptor isoforms in primary NSCLC was largely unknown.

In this study, we leveraged recently available RNA-seq data generated from well-characterized primary NSCLC tumors by TCGA to evaluate mRNA expression of the splice variants IR-A and IR-B. This analysis was extended to include additional NSCLC samples beyond those available from TCGA, as well as additional tumor types. In summary, in all NSCLC datasets evaluated, we observed down-regulation of IR-B mRNA expression with an associated increase in the IR-A/IR-B mRNA ratio in a subpopulation of patients. We also found a significant increase in the prevalence of patients with HIR in BRCA, COAD, KIRC, KIRP, LIHC, and UCEC compared to normal tissues, with the prevalence of HIR in brain tumors (both GMB and LGG) greater than 93%. These findings are important because they suggest that many cancer types experience alterations in the INSR pathway, which may be important for disease pathogenesis. Although we observed differences in the mRNA expression levels of IR-A in the TCGA dataset compared with our internal data, it may be due to overall differences in sensitivity and specificity between the platforms for IR-A quantitation and/or heterogeneity within tumor samples between our test panels and TGCA. Additional work will need to be done to get a fuller understanding of the role of IR-A and IR-B expression in NSCLC and other cancer types.

Given the role of the INSR in glucose metabolism, we postulate that the change of IR-A/IR-B mRNA ratio in tissues could be associated with differences in metabolism in tumor tissue compared to normal tissue. It has been shown previously that glucose metabolism in cancer cells is altered from normal oxidative phosphorylation to glycolysis [16], such that tumor cells take up much more glucose and mainly process it through aerobic glycolysis rather than oxidative phosphorylation i.e. the ‘Warburg effect’ , [17]. This metabolic switch emphasizes the production of intermediates necessary for tumor growth and division and has been shown to be regulated by oncogenes and tumor suppressor genes in a number of key cancer growth pathways [18]. Alterations in the IR-A/IR-B mRNA ratio in NSCLC might be related to the Warburg effect in tumors. The down regulation of IR-B could be a negative feedback from cancer cells in response to the high glucose intake, thereby decreasing the Warburg effect in cancer cells. This is suggested by our observations that cancers with higher IR-A/IR-B mRNA ratio have higher mRNA expression levels of genes involved in oxidative phosphorylation, as well as a less cancerous phenotype and greater survival in LUSC. At the same time, since the IRA has high affinity to IGF2, the higher IR-A/IR-B mRNA ratio could push cells to depend more on IGF2 signaling through IR-A [19], resulting in increased proliferation signaling. The concentration of IGF2 in the microenvironment of a particular tumor type may be critical to influence the effect of the increased IR-A/IR-B mRNA ratio. For example, for tumor types without high IGF2, increased IR-A/IR-B mRNA ratio may be of benefit to the patients due to the lack of proliferation signaling through IR-A.

To further understand the molecular characteristics associated with the changes in the IR-A/IR-B mRNA ratio in NSCLC, we analyzed the differentially expressed genes between the HIR and LIR groups. We found in both LUAD and LUSC, genes involved in extracellular matrix (ECM) interactions such as collagen type I, V, VI, X; integrin, alpha 8, 11; and fibronectin type III domain containing 1 are significantly down regulated in patients with HIR (P < 0.01). The enzymes involved in ECM remodeling such as metallopeptidases cartilage oligomeric matrix protein, and matrix-remodeling associated 5 are also significantly down regulated (P < 0.01). All these genes are part of EMT signatures [20], where EMT is a process by which epithelial cells lose their cell polarity and cell-cell adhesion while gaining migratory and invasive properties to become mesenchymal cells [21,22]. In NSCLC cancer specifically, EMT has been associated with EGF receptor inhibitor resistance and worse clinical outcome [20]. ECM/integrin signaling has also been demonstrated to provide a survival advantage to various cancer cell types against chemotherapeutic drugs and antibody therapy [23]. The association of HIR with decreased EMT signatures described in this manuscript suggests that HIR patients have less invasive and metastatic lung cancers, which is consistent with the observed association between HIR and increased survival. Based on our previous data in breast cancer, these results were somewhat unexpected; however, they reveal that the relevance of the IR-A/IR-B mRNA ratio as a cancer biomarker has to be evaluated for each tumor type. The complex relationship between altered INSR isoforms and cancer prognosis may help explain the challenges associated with targeting the IGF1R pathway in NSCLC and other cancers. It is also important to note that there are currently no methods available to quantify the protein level of IRA and IRB. These data could be helpful for subsequent evaluation of the clinical utility of IRA/IRB mRNA ratio in caner.

## Conclusion

HIR was commonly observed in multiple solid tumors surveyed in this study, although the functional importance of these increased ratios likely differ between these tumor types. Since both IR-A and IR-B are regulated by insulin, IGF1 and IGF2 through autocrine and/or paracrine mechanisms, the microenvironment of the cancer site may also contribute to the relationship of HIR with clinical prognosis and patient survival. Therefore, the exact impact of the increase in IR-A/IR-B mRNA ratio in the clinical course of specific cancer types needs to be carefully evaluated before initiation of any intervention, especially those targeting the IGF pathway. Blocking altered metabolic signaling pathways through the IGF axis could benefit patients of one type of cancer but may be less likely to benefit patients with other cancer types. Accordingly, the knowledge that the IR-A/IR-B mRNA ratio is altered across multiple cancer indications is the foundation for continued efforts to characterize the effect of this alteration on cancer progression and therapeutic response in order to achieve the most benefit.

## Competing interests

WZ, KS ,CM, PB KR, BWH YY, JH are employees of Medimmune. ZD, XY, GZ, YG are employees of AstraZeneca and own the stocks of AstraZeneca. Other authors declare that they have no competing interests.

## Authors’ contributions

LJ, WZ integrated the data and completed the manuscript as a major contributor; JH, YY conceptualized the report, integrated the data, write the manuscript, and gave final approval to the manuscript as a corresponding author; WZ and BWH carried out statistical evaluation of RNAseq expressions in TGCA data, KS, CM PB, evaluated the mRNA expressions of IRA and IRB in cDNA samples of NSCLC and integrated the data. LJ identified primary NSCLC patients provide clinical information of these patients, LJ, ZD, XY, GZ, YG carried out the histopathological examinations in primary NSCLC and evaluated the mRNA expressions of IRA and IRB in primary NSCLC samples and integrated the data, KSR integrated and interpreted the data. Furthermore, all authors contributed towards the conceptualization, writing, reading, and approval of the final manuscript.

## Pre-publication history

The pre-publication history for this paper can be accessed here:

http://www.biomedcentral.com/1471-2407/14/131/prepub
